# Inverse association between serum bilirubin level and testosterone deficiency in middle-aged and older men

**DOI:** 10.1038/s41598-021-87220-z

**Published:** 2021-04-13

**Authors:** Hye-Min Park, Haeyoung Kim, Hye Sun Lee, Yong-Jae Lee

**Affiliations:** 1 Department of Family Medicine, Chaum Medical Checkup Center Samseongdong Branch, Cha University, Seoul, Republic of Korea; 2 Department of Medicine, Graduate School of Medicine, Yonsei University, Seoul, Republic of Korea; 3 Department of Integrative Medicine, Major in Digital Healthcare, Yonsei University College of Medicine, Seoul, Republic of Korea; 4 Biostatistics Collaboration Unit, Department of Research Affairs, Yonsei University College of Medicine, Seoul, Republic of Korea; 5 Department of Family medicine, Yonsei University College of Medicine, Seoul, Republic of Korea

**Keywords:** Biomarkers, Endocrinology, Medical research

## Abstract

Low serum bilirubin levels have been associated with increased risk of cardiovascular disease (CVD) and metabolic syndrome. Testosterone deficiency could also contribute to increased risk of CVD and metabolic syndrome. Therefore, this study aimed to examine the relationship between serum bilirubin level and testosterone deficiency in 1284 Korean men aged 45 to 70 years. Serum bilirubin level was categorized into quartiles: Q1 ≤ 0.7, Q2 0.8–0.9, Q3 1.0–1.1, and Q4 ≥ 1.2 mg/dL. Testosterone deficiency was defined as level less than 8.0 nmol/L, as suggested by the position statement of International Society of Andrology. The overall prevalence of testosterone deficiency was 5.8% and significantly decreased with the quartiles from Q1 to Q4. Compared with the referent fourth quartile (serum bilirubin ≥ 1.2 mg/dL), the ORs (95% CIs) for testosterone deficiency was 2.29 (1.04–4.94) for the first quartile after adjusting for age, fasting glucose, triglyceride, HDL-cholesterol, leukocyte count, hemoglobin, smoking status, and alcohol intake. We found inversely graded associations of serum bilirubin level with testosterone deficiency. These findings suggest that low bilirubin level may be interpreted as a state of testosterone deficiency in middle-aged and older men.

## Introduction

Testosterone level in men is well documented to remain stable until around 40 years of age and then to decline at about 1% per year as a natural result of aging^1^. Male hypogonadism is a condition in middle-aged and older men characterized by low testosterone level with clinical symptoms including sexual dysfunction, depressed mood, lack of muscle mass, abdominal obesity, and deteriorated quality of life^2,3^. Approximately 2–6% of men aged over 65 years will have symptoms of hypogonadism^4^. Accumulating epidemiological evidence shows that testosterone deficiency contributes to increase risk of metabolic syndrome and cardiovascular disease (CVD)^5^, which are highly linked to chronic low-grade inflammation. Hence, early identification of individuals at higher risk for testosterone deficiency is important for male health and quality of life.

Bilirubin is made from biliverdin by the work through heme oxygenase and biliverdin reductase and is an end product of heme catabolism^6^. Serum bilirubin level has been regarded as an indicator of hepatobiliary dysfunction or hemolysis^7^, but all the evidence that has piled up suggests that bilirubin is a potent antioxidant and endogenous cytoprotective agent in the progress of cardiometabolic diseases^8^. Previous studies show that low level of serum bilirubin is relevant to increased risk of metabolic syndrome, carotid atherosclerosis, and coronary artery disease^9–11^.

Since low levels of both testosterone and bilirubin are associated with elevated risk of cardiometabolic diseases, we inferred a possible association between testosterone levels and serum bilirubin. Therefore, this study aimed to examine the relationship between serum bilirubin level and testosterone deficiency in 1284 Korean men aged 45–70 years.

## Methods

### Study population

We reviewed the medical records of 1401 participants aged ≥ 45 years retrospectively who had a medical examination between November 2012 and July 2013 at the Health Promotion Center of Gangnam Severance Hospital in Seoul, Korea. The individuals visited the health promotion center willingly to regularly check their health condition. Written informed consent was obtained from all participants. This study was authorized by the Institutional Review Board of Yonsei University College of Medicine, Seoul, Korea (approval No. 3-2019-0319) and was managed in according to the ethical principles of the Declaration of Helsinki. We excluded men who met at least one of the following criteria: individuals with a history of hepatobiliary disease, positive test for hepatitis C antigens or hepatitis B antibodies, a history of exogenous testosterone therapy, above twice the upper normal limit for aspartate aminotransferase (AST) or alanine aminotransferase (ALT), total bilirubin levels of more than 3.0 mg/dL, hemoglobin level of less than 13 g/dL, not fasting for 12 h before testing or missing data. After applying the exclusion criteria, the final analysis included 1284 participants.

### Data collection

Each participant was asked to fill in a questionnaire about his medical history and lifestyle. Self-reported alcohol consumption, physical activity, and cigarette smoking were determined from the questionnaires. Smoking status was classified as current smoker, ex-smoker, and non-smoker. Alcohol drinking was assessed into consumption ≥ twice a week. Regular exercise was considered as more than 30 min of aerobic exercise ≥ three times a week. Body mass and height were measured to the nearest 0.1 kg and 0.1 cm, respectively, in light indoor clothing without shoes. Body mass index (BMI) was calculated as weight in kilograms divided by square of height in meters (kg/m^2^). Systolic blood pressure (SBP) and diastolic blood pressure (DBP) were measured using a standard mercury sphygmomanometer (Baumanometer, W.A. Baum Co Inc., Copiague, NY, USA) and the patient’s right arm. After a 12-h overnight fast, all blood samples were taken from the antecubital vein. Testosterone concentrations were determined using an electrochemiluminescence assay with a Modular Analytics E170 system (Roche Diagnostic Systems, Basel, Switzerland). The reproducibility was determined by calculating the intra- and inter-assay coefficients of variation (CV) as follow: [% CV = (SD/mean) * 100]. For intra-assay CV, the same serum was assayed three times in duplicate within the same assay. The inter-assay reproducibility was assessed by analyzing six times the consecutive assays in duplicate^12^. The intra-assay and inter-assay CVs for testosterone were 3.2% and 3.3%, respectively. Fasting glucose, triglycerides, high density lipoprotein (HDL) cholesterol, AST, ALT, and total bilirubin were quantified with enzymatic methods using a chemistry analyzer (Hitachi 7600, Hitachi Co., Tokyo, Japan). Leukocyte count was measured using an automated blood cell counter (ADVIA 120, Bayer, NY, USA).

Hypertension was defined as a systolic blood pressure ≥ 140 mmHg, diastolic blood pressure ≥ 90 mmHg, or current use of hypertension medication^13^. Type 2 diabetes was defined as current use of diabetes medication or fasting plasma glucose ≥ 126 mg/dL^14^. Dyslipidemia was set as triglyceride ≥ 150 mg/dL, HDL-cholesterol < 40 mg/dL, or use of dyslipidemia medications at present. The modified National Cholesterol Education Program Adult Treatment Panel III (NCEP-ATP III) released in 2001 was applied to designate metabolic syndrome^15^. According to the position statement of the American College of Endocrinology, we defined obesity as BMI ≥ 25 kg/m^2^, since waist circumference was not measured^16^. Thus, metabolic syndrome was defined by the existence of three or more of risk factors as follows: obesity with BMI ≥ 25 kg/m^2^, elevated fasting plasma glucose ≥ 126 mg/dL or current use of diabetes medication, high systolic blood pressure ≥ 130 mmHg, diastolic blood pressure ≥ 85 mmHg, anti-hypertensive drugs, low HDL cholesterol < 40 mg/ dL and high triglycerides ≥ 150 mg/dL.

### Statistical analysis

Normal distribution was assessed with determination of skewness by Kolmogorov–Smirnov test. ALT, AST, serum triglyceride levels had skewed distributions, therefore these variables were indicated as median with interquartile range (IQR) in descriptive analysis and log-transformed preceding to simple correlation and multiple logistic regression analysis. Serum bilirubin level was divided into quartiles: Q1 ≤ 0.7, Q2 0.8–0.9, Q3 1.0–1.1, and Q4 ≥ 1.2 mg/dL. The clinical characteristics of the study population according to serum bilirubin quartile were independently compared using one-way analysis of variance (ANOVA) or Kruskal–Wallis test for continuous variables in accordance with the normality of distributions and chi-square test for categorical variables. Continuous data are exhibited as mean (standard deviation [SD]) or median (IQR), and categorical data are marked as frequency. Testosterone deficiency was defined as level less than 8.0 nmol/L, as proposed by the announcement of the International Society of Andrology (ISA)^17,18^. The odds ratios (ORs) and 95% confidence intervals (95% CIs) for testosterone deficiency were calculated after adjusting for confounding variables over serum bilirubin quartiles using multiple logistic regression analysis. We have included the confounding variables in the logistic regression analysis model considering the commonly performed statistical principles to include clinically established important risk factors and statistically significant variables in the simple regression analysis. All analyses were carried out using SAS statistical software (version 9.4; SAS Institute Inc., Cary, NC, USA). All statistical tests were two-sided, and statistical significance was determined at *P* < 0.05.

### Informed consent

Informed consent was obtained from all the participants in the current study.

## Results

Table1 shows the clinical characteristics of the study population according to serum bilirubin quartiles. Leukocyte count and fasting plasma glucose level were lowest, while HDL- cholesterol level was highest in the fourth quartile of bilirubin concentration. The proportions of current smoking was highest in the first quartile of serum bilirubin. The prevalence of metabolic syndrome, dyslipidemia, and type 2 diabetes decreased with increasing serum bilirubin quartile.

**Table 1:** Clinical characteristics of the study population according to serum bilirubin quartiles.

	Serum bilirubin quartiles (mg/dL)					
	Q1 (≤ 0.7)	Q2 (0.8–0.9)	Q3 (1.0–1.1)	Q4 (≥ 1.2)	*P* value	Post hoc
n	275	356	302	351		
Age (years)	57.3 (8.1)	56.4 (7.3)	54.7 (7.1)	55.2 (7.3)	0.001	b,c,d
Body mass index (kg/m^2^)	24.9 (2.6)	24.6 (2.5)	24.6 (2.5)	24.4 (2.5)	0.197	
Systolic BP (mmHg)	126.4 (15.0)	127.3 (15.2)	127.2 (17.0)	127.4 (16.8)	0.859	
Diastolic BP (mmHg)	78.9 (8.9)	79.6 (8.7)	80.2 (9.8)	79.8 (9.6)	0.339	
Mean arterial pressure (mmHg)	94.7 (10.4)	95.6 (10.3)	95.9 (11.5)	95.7 (11.5)	0.577	
Hemoglobin (g/dL)	15.0 (0.9)	15.1 (1.0)	15.4 (1.1)	15.6 (1.0)	< 0.001	b,c,d,e
Hematocrit (%)	44.2 (2.6)	44.5 (2.8)	45.0 (3.1)	45.3 (3.0)	< 0.001	b,c,e
Fasting glucose (mg/dL)	105.6 (27.0)	102.8 (23.3)	100.6 (22.3)	99.5 (17.1)	0.006	b,c,
Triglyceride (mg/dL)	127 (93–175)	113 (81–158)	112 (85–169)	109 (80–149)	0.014	c
HDL-cholesterol (mg/dL)	44.0 (10.9)	47.1 (11.2)	47.4 (11.6)	48.4 (10.8)	< 0.001	a,b,c
Aspartate aminotransferase (U/L)	22 (18–26)	22 (19–26)	22 (18–27)	22 (19–26)	0.963	
Alanine aminotransferase (U/L)	23 (18–32)	23 (19–32)	24 (18–33)	22 (18–30)	0.703	
Leukocyte (cells/μL)	6590 (1740)	6217 (1801)	6019 (1571)	5984 (1674)	< 0.001	a,b,c
Current smoking (%)	43.6	31.7	28.7	22.1	< 0.001	b,c,d
Alcohol drinking (%)^g^	45.6	52.2	53.5	44.5	0.053	
Regular exercise (%)^h^	40.5	43.1	42.8	45.8	0.632	
Hypertension (%)^i^	45.5	46.9	43.4	40.5	0.348	
Type 2 diabetes (%)^j^	17.8	14.6	11.1	10.9	0.042	c
Dyslipidemia (%)^k^	35.6	30.4	28.8	24.4	0.024	c
Metabolic syndrome (%)	40.3	36.8	32.1	30.2	< 0.001	b,c

*BP* blood pressure, *HDL* high density lipoprotein, *NA* not applicable. Data are expressed as the mean (SD), median (IQR), or percentage. P-values were calculated using 1-way ANOVA test, Kruskal–Wallis test, or chi-square test. The differences of mean, median and proportion between groups were determined using post hoc analysis of ANOVA, Kruskal–Wallis test and chi-squared test using Bonferroni corrections.

^a^Q1 versus Q2, ^b^Q1 versus Q3, ^c^Q1 versus Q4, ^d^Q2 versus Q3, ^e^Q2 versus Q4, ^f^Q3 versus Q4, ^g^Alcohol drinking ≥ twice/week. ^h^Regular exercise ≥ three times/week. ^i^Hypertension was defined as a systolic blood pressure ≥ 140 mmHg, diastolic blood pressure ≥ 90 mmHg, or the current use of hypertension medication. ^j^Type 2 diabetes was defined as a fasting plasma glucose ≥ 126 mg/dL or the current use of diabetes medication. ^k^Dyslipidemia was defined as triglyceride ≥ 150 mg/dL or HDL-cholesterol < 40 mg/dL for men or the current use of dyslipidemia medications.

**Figure 1:**
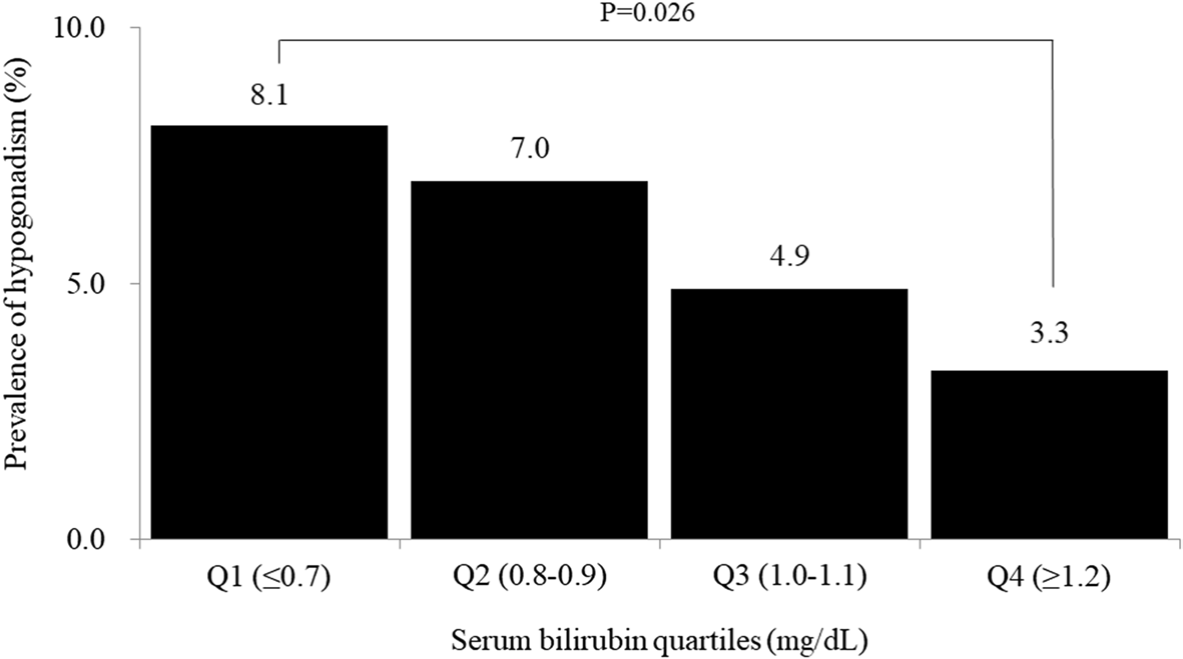
Prevalence of testosterone deficiency according to serum bilirubin quartiles (P value was calculated by ANOVA test).

Figure 1 presents the prevalence of subjects with testosterone deficiency in each bilirubin quartile. The overall prevalence of testosterone deficiency was 5.8% and significantly decreased in accordance with serum bilirubin quartile (Q1 8.1%, Q2 7.0%, Q3 4.9%, and Q4 3.3%) (*P* = 0.026).

Table 2 indicates the results of multiple logistic regression analysis to assess the association between serum bilirubin level and testosterone deficiency. Compared with the referent fourth quartile (serum bilirubin ≥ 1.2 mg/dL), the ORs (95% CIs) for testosterone deficiency was 2.29 (1.04–4.94) for the first quartile after adjusting for age, fasting glucose, triglyceride, HDL-cholesterol, leukocyte count, hemoglobin, smoking status, and alcohol intake.

**Table 2:** Odds ratios and 95% confidence for testosterone deficiency according to serum bilirubin quartiles.

	Serum bilirubin quartiles (mg/dL)			
	Q1 (≤ 0.7)	Q2 (0.8–0.9)	Q3 (1.0–1.1)	Q4 (≥ 1.2)
Model 1^a^	2.15 (1.07-4.92)	1.99 (0.92-4.52)	1.27 (0.60-2.67)	1.00
Model 2^b^	2.23 (1.06-4.98)	1.98 (0.95-4.50)	1.24 (0.59-2.64)	1.00
Model 3^c^	2.29 (1.04-4.94)	1.99 (0.94-4.39)	1.32 (0.73-3.60)	1.00

^a^Adjusted for age.

^b^Adjusted for age, fasting plasma glucose, and triglyceride.

^c^Adjusted for age, fasting plasma glucose, triglyceride, HDL-cholesterol, leukocyte count, hemoglobin, smoking status, and alcohol intake.

## Discussion

In this cross-sectional study, serum bilirubin level was independently and inversely associated with testosterone deficiency in a dose–response way after adjusting for potential confounding variables. To date, it has been rarely investigated on the association between serum bilirubin level and testosterone deficiency. The plausible explanations may be offered to explain the inverse relationship between serum bilirubin level and testosterone deficiency.

First, low levels of testosterone and bilirubin are closely interrelated to oxidative stress and chronic low-grade inflammation. In our study, serum bilirubin level was inversely associated with leukocyte count, a nonspecific marker of systemic inflammation. Hwang et al. also reported that CRP level decreased with increasing bilirubin quartiles among apparently healthy adults^19^. Both animal and human studies have documented that bilirubin could serve as a potent lipid chain-breaking antioxidant under physiological concentrations, inhibiting the formation of atherogenic plaques^20,21^. Given the antioxidant capacity of bilirubin, epidemiological links between low serum bilirubin level and increased risk for atherosclerotic CVD and the severity of CAD have been shown in cross-sectional and longitudinal studies^22^. Among 4303 participants aged ≥ 60 years in the NHANES, a low total bilirubin level of 0.1–0.4 mg/dL was associated with higher mortality risk^23^. English et al. found statistically significant lower levels of testosterone in patients with catheterization-proven CAD compared with control groups^24^. In addition, mildly elevated unconjugated bilirubin reduces the risk of CVD in individuals with Gilbert’s syndrome^25^. Likewise, extended evidence also implies that chronic low-grade inflammation is closely involved in the pathogenesis of male hypogonadism^26^. Pro-inflammatory cytokines such as tumor necrosis factor-α and interleukin-6 are released from adipose tissues in testosterone deficiency, which leads to a subclinical inflammatory condition^27^. A recent study suggests that low testosterone levels may be interpreted as a state of low-grade inflammation. Park et al. revealed an inverse associations of testosterone levels with leukocyte counts in Korean adults^26^.

Second, low levels of testosterone and bilirubin are closely related to insulin resistance and metabolic abnormalities^28,29^. Recent epidemiological studies have shown that there is an inverse association of serum bilirubin and testosterone levels with cardiometabolic disturbances, such as obesity, hypertension, type 2 diabetes, and metabolic syndrome. In a prospective study of 468 Taiwanese middle-aged men, a higher bilirubin level was associated with a reduced future risk of metabolic syndrome particularly in nonsmoking men^30^. Our study also found a significant difference in the prevalence of type 2 diabetes, dyslipidemia, and metabolic syndrome according to serum bilirubin quartiles. A low testosterone level could be a result of insulin resistance through increasing visceral adiposity and decreasing skeletal muscle^31^. Low testosterone also increases lipoprotein lipase activity, leading to increase triglyceride uptake in central fat depots^32^, insulin resistance and metabolic syndrome. Thus, increased abdominal visceral fat and low testosterone levels have bidirectional interactions. Previous studies have demonstrated that hypercholesterolemia, glycemic control, visceral adiposity and insulin resistance in hypogonadal men with type 2 diabetes or metabolic syndrome were improved through testosterone replacement therapy^33,34^. Aslo, Wickramatilake et al. showed a positive association of testosterone with HDL-cholesterol level in angiographically confirmed CAD in men^35^.

This study had some limitations to its interpretation. First, we used a cross-sectional design and were not able to prove a causal relationship between serum bilirubin and testosterone deficiency. The results may mirror reverse causality and a bidirectional relationship in the association between these variables. Therefore, further large-scale with a long follow-up period, prospective studies are warranted to establish whether there is a cause-and-effect relationship between serum bilirubin and testosterone deficiency. Second, the study population might not represent the general population because the study participants were volunteers visiting a single health promotion center to screen their health condition and seemed to be a little bit healthier than most community-based cohorts. Third, only one measurement of bilirubin and testosterone levels from a baseline examination was included in the analyses. Fourth, the definition of type 2 diabetes does not meet the criteria of American Diabetes Association, since any information about HbA1c and 2-h plasma glucose levels were not collected from the dataset. Finally, the cut-off point for testosterone deficiency in the present study was defined as < 8.0 nmol/L as proposed by the announcement of the ISA. Men with levels between 8–12 nmol/L also may be testosterone deficiency, however testosterone deficiency symptoms and free testosterone levels should also be considered in this range of serum total testosterone. Unfortunately, we could not collect further information on testosterone deficiency symptoms and free testosterone levels from the initial dataset.

In conclusion, we found an inversely graded association of serum bilirubin level with testosterone deficiency. In conclusion, we found an inversely graded association of serum bilirubin level with testosterone deficiency and CVD risk factors. These findings suggest that low serum bilirubin level may be assumed as a state of testosterone deficiency and CVD risk in middle-aged and older men.

## Data Availability

The datasets in this study are available from the corresponding author on reasonable request.
